# Deep Learning in Oral Hygiene: Automated Dental Plaque Detection via YOLO Frameworks and Quantification Using the O’Leary Index

**DOI:** 10.3390/diagnostics15020231

**Published:** 2025-01-20

**Authors:** Alfonso Ramírez-Pedraza, Sebastián Salazar-Colores, Crystel Cardenas-Valle, Juan Terven, José-Joel González-Barbosa, Francisco-Javier Ornelas-Rodriguez, Juan-Bautista Hurtado-Ramos, Raymundo Ramirez-Pedraza, Diana-Margarita Córdova-Esparza, Julio-Alejandro Romero-González

**Affiliations:** 1 Centro de Investigación en Ciencia Aplicada y Tecnología Avanzada, Instituto Politécnico Nacional, Querétaro 76090, Mexico or pedro.ramirez@secihti.mx (A.R.-P.); jgonzalezba@ipn.mx (J.-J.G.-B.); fornelasr@ipn.mx (F.-J.O.-R.); jbautistah@ipn.mx (J.-B.H.-R.); 2 Secretaría de Ciencia, Humanidades, Tecnología e Innovación SECIHTI, IxM, Alvaro Obregón 03940, Mexico; 3 IA, Centro de Investigaciones en Óptica A.C., Loma del Bosque 115, León 37150, Mexico; sebastian.salazar@cio.mx; 4 Clinica Lurviva, I. Allende, Apaseo el Grande 38160, Mexico; crystelcardenas6@gmail.com; 5 Facultad de Contaduria y Administración, Universidad Autónoma de Querétaro, Querétaro 76017, Mexico; raymundo.ramirez@uaq.mx; 6 Facultad de Informática, Universidad Autónoma de Querétaro, Querétaro 76230, Mexico; diana.cordova@uaq.mx (D.-M.C.-E.); julio.romero@uaq.mx (J.-A.R.-G.)

**Keywords:** deep learning, model, plaque, dentobacteria, O’Leary index

## Abstract

**Background**: Oral diseases such as caries, gingivitis, and periodontitis are highly prevalent worldwide and often arise from plaque. This study focuses on detecting three plaque stages—new, mature, and over-mature—using state-of-the-art YOLO architectures to enhance early intervention and reduce reliance on manual visual assessments. **Methods**: We compiled a dataset of 531 RGB images from 177 individuals, captured via multiple mobile devices. Each sample was treated with disclosing gel to highlight plaque types, then preprocessed for lighting and color normalization. YOLOv9, YOLOv10, and YOLOv11, in various scales, were trained to detect plaque categories, and their performance was evaluated using precision, recall, and mean Average Precision (mAP@50). **Results**: Among the tested models, YOLOv11m achieved the highest mAP@50 (0.713), displaying superior detection of over-mature plaque. Across all YOLO variants, older plaque was generally easier to detect than newer plaque, which can blend with gingival tissue. Applying the O’Leary index indicated that over half of the study population exhibited severe plaque levels. **Conclusions**: Our findings demonstrate the feasibility of automated plaque detection with advanced YOLO models in varied imaging conditions. This approach offers potential to optimize clinical workflows, support early diagnoses, and mitigate oral health burdens in low-resource communities.

## 1. Introduction

Oral diseases such as dental caries, gingivitis, periodontitis, edentulism, cleft lip and palate, orofacial clefts, noma, oral trauma, and oral cancers are largely preventable and can often be treated in their early stages.

The World Health Organization (WHO), in its Global Oral Health Status Report (2022) [1], estimated that nearly 3.5 billion people worldwide suffer from some form of oral disease, representing 45% of the global population, many of which are linked to dental biofilm. It is estimated that two billion people have dental caries, including 514 million children, making it the most common condition. The WHO also highlights that three out of four people live in middle-income countries. Most low- and middle-income countries lack adequate prevention and treatment services for oral health conditions.

Dental plaque, also known as dental biofilm, is responsible for the occurrence of many oral diseases, such as tartar, cavities, gingivitis, periodontitis, or halitosis. Plaque forms from foods consumed, and saliva feeds the bacteria living in the mouth, creating a sticky and colorless film. Sugars turn into acids that destroy the tooth.

The Ministry of Health (SS) of Mexico states that 90% of the population has an oral disease; cavities, prevalent in childhood and in adulthood, periodontal diseases, malocclusion, inadequate dental positioning, and temporomandibular dysfunctions mainly predominate. In older adults, tooth loss is predominant. These diseases represent a significant burden on the health sector and are responsible for deaths that, to a large extent, could be prevented through early interventions.

The formation of dental plaque is a dynamic process influenced by time and oral hygiene habits. It begins shortly after brushing, when an acquired film of salivary proteins covers the tooth surface. This film is colonized by bacteria within the first few hours, resulting in the formation of new plaque, as we refer to it in our article. In the absence of proper hygiene, this plaque evolves over two to three weeks, developing into mature plaque, characterized by greater bacterial complexity. If not removed, the plaque can mineralize due to the deposition of minerals present in saliva, forming a hardened plaque, referred to in our article as over-mature plaque, commonly known as tartar or dental calculus. These stages reflect the natural progression of the oral biofilm and its potential impact on oral health.

Identifying the three types of biofilm allows for a more accessible assessment of caries risk by associating bacterial colonization with the acidogenic properties of dental plaque. In addition to being the main cause of caries, plaque is associated with diseases such as gingivitis, periodontitis, halitosis, and systemic complications including bacterial endocarditis, diabetes, respiratory infections, and pregnancy-related risks.

The objective of this study is to identify the three types of dental plaque: new, mature, and over-mature. Our data include patients of the central region of Mexico in the cities of Celaya, Apaseos, Comonfort, Juventino Rosas, Silao, and León. Our goal is to present a methodology that allows simulating dental processes using an object detector based on convolutional neural networks.

### 1.1. Use of YOLO in Object Detection

Object detection algorithms have evolved significantly in recent years, and the YOLO family (‘You Only Look Once’) has distinguished itself for its real-time capabilities and high accuracy. YOLO was initially introduced to perform single-shot detection, treating object localization and classification as a single regression problem [2]. In successive releases, the YOLO architecture has undergone substantial improvements. In this paper, we evaluate the latest three iterations of YOLO to detect dental plaque as described below.

#### 1.1.1. YOLOv9

The YOLOv9 [3] architecture addresses the bottleneck problem where data are lost across different layers. By utilizing Programmable Gradient Information (PGI), it ensures reliable gradient backpropagation and efficient gradient flow. This version achieves a powerful yet lightweight architecture by efficiently managing computational resources while maintaining precision and speed.

**Figure 1:**
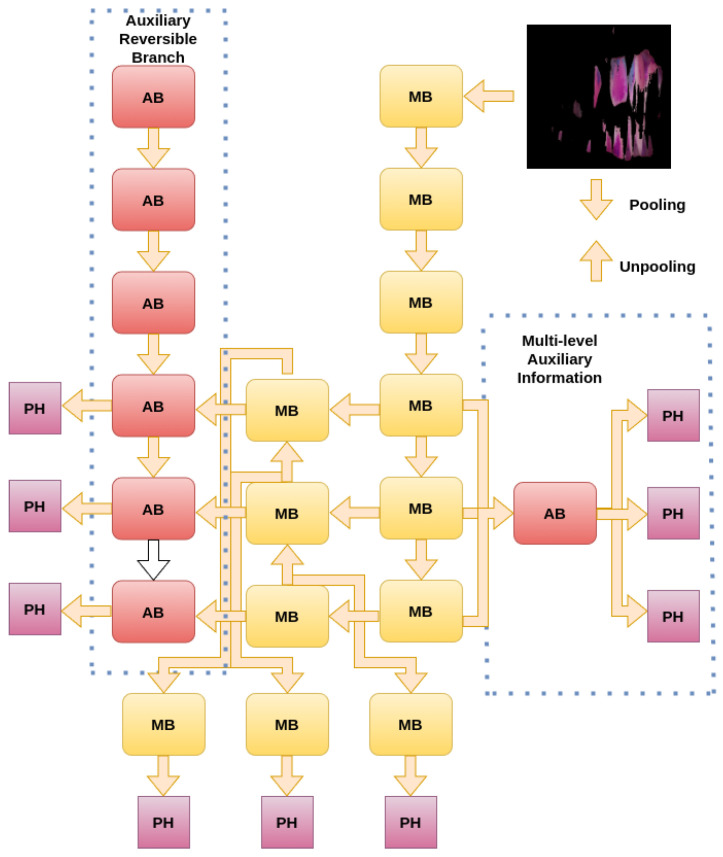
YOLOv9 Architecture: It is composed of three main components: the reversible auxiliary branch (optimizing gradient flow), the primary prediction network (responsible for feature extraction), and the multi-level auxiliary information (aggregating and propagating features across stages). The prediction heads (PHs) generate the final outputs, efficiently integrating features through pooling and unpooling operations.

YOLOv9 consists of three main components. First, the backbone is based on CSPNet (Cross-Stage Partial Networks) principles. Second, a reversible auxiliary branch enhances the propagation of PGI (Programmable Gradient Information). Third, the detection head divides the image into grids and simultaneously predicts classes, improving Non-Maximum Suppression (NMS). Lastly, it incorporates scalable layers or models, including variants t, s, m, c, and e. Figure 1 illustrates the YOLOv9 architecture.

#### 1.1.2. YOLOv10

A significant change in YOLOv10 [4] compared to previous models is the dual coherent strategy, which eliminates the need for Non-Maximum Suppression (NMS) during inference. It features a holistic design focused on precision and efficiency, reducing computational overhead. Additionally, it minimizes inference latency while maintaining competitive performance by combining one-to-one and one-to-many label assignments.

**Figure 2:**
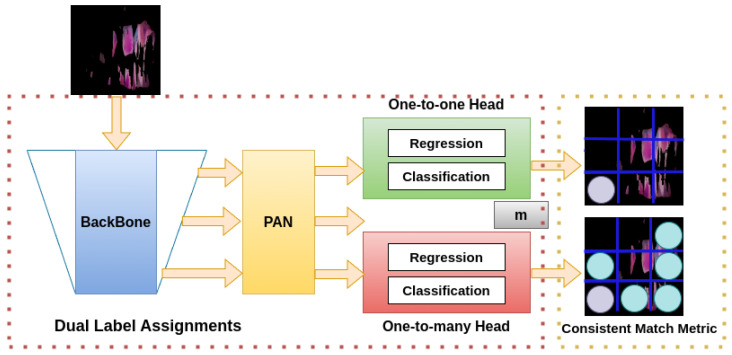
YOLOv10 architecture. The structure has three components: backbone, PAN (Path Aggregation Network), and prediction heads. The backbone extracts features from the input image, and the PAN efficiently aggregates and propagates features across network levels. YOLOv10 introduces Dual Label Assignments with two prediction heads: One-to-One Head and One-to-Many Head, for regression (bounding boxes) and classification tasks. A Consistent Match Metric optimizes label assignment to enhance prediction accuracy across scales and conditions.

YOLOv10 consists of three main components. First, the PAN backbone and prediction heads (Path Aggregation Network) handle initial feature extraction, and PAN facilitates efficient feature aggregation and propagation. Second, Dual Label Assignments enable two prediction processes, one-to-one and one-to-many, to perform regression and classification tasks. Finally, to optimize label assignment and enhance accuracy, the Consistent Match Metric is applied under various conditions and scales. Figure 2 presents a summarized architecture of YOLOv10.

#### 1.1.3. YOLOv11

YOLOv11 [5] improves on YOLOv8 by optimizing parameters to achieve superior detection performance.

**Figure 3:**
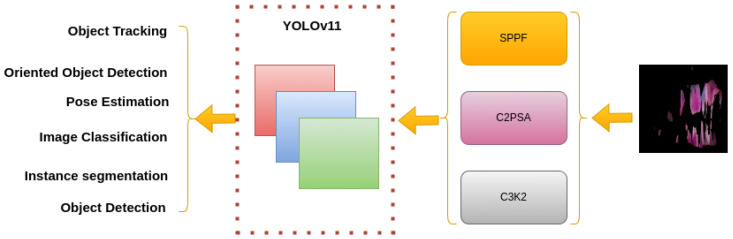
YOLOv11 architecture: YOLOv11 is an efficient model for tasks like object detection, tracking, oriented detection, pose estimation, image classification, and instance segmentation. It uses enhanced blocks like SPPF, C2PSA, and C3K2 for better feature extraction, spatial attention, and efficiency.

YOLOv11 introduces significant improvements in feature extraction, efficiency, and multitasking capabilities. It also features architectural innovations, such as improved backbone and neck structures, while improving performance on object detection, segmentation, and pose estimation tasks. Figure 3 illustrates the summarized architecture of YOLOv11.

These latest YOLO variants have shown remarkable versatility in a wide range of detection scenarios. In our work, we leverage YOLOv9, YOLOv10, and YOLOv11 to automatically detect three types of dental plaque (new, mature, and over-mature). This automation aims to improve diagnostic accuracy in dentistry while reducing the time and potential subjectivity associated with manual or purely visual examinations.

This paper is structured as follows. Section 2 describes the related works. Section 3 presents our methodology, followed by Section 4 presenting our results. We conclude with a discussion and summary in Section 5.

## 2. Related Works

Traditional methods for detecting dental plaque, while usually effective, are limited by their subjectivity and time-consuming nature. Introducing new technologies into the field has the potential to streamline detection procedures, making them both more efficient and objective. In this section, we review related works and highlight how our approach contrasts with and contributes to current research on automatic dental plaque detection.

AI advances have significantly impacted a wide range of scientific and medical fields, including dentistry. Recent studies emphasize how artificial intelligence is revolutionizing endodontics, oral radiology, and orthodontics, among other specialties [6]. This growing body of research demonstrates the role of AI in enhancing diagnostic precision, optimizing treatment planning, and promoting the discovery of innovative therapies. By improving patient care standards, AI-based methods have shown superiority over conventional techniques, allowing more personalized and efficient treatments.

The increasing demand for precise diagnostics spurs the development and adoption of computational tools to assist in medical diagnosis [7]. Vision algorithms and machine learning (ML) models are rapidly altering workflows in many dental specializations. Their superiority over conventional techniques is evidenced in fields such as oral radiology, pediatric dentistry, restorative dentistry, endodontics, prosthodontics, orthodontics, and periodontics [6]. In this context, a comprehensive study of AI in dentistry reveals how diagnostic automation, detection of dental disease by X-rays (e.g., from companies such as KaVo Dental GmbH, Biberach an der Riss, Germany, or Carestream Dental LLC, Atlanta, GA, USA), and improvements in data quality and AI tools converge to transform standard practices [8].

Early detection of dental pathologies holds considerable value for patient outcomes. Previous work has shown how automation can aid in cephalometric tracking, growth estimation, and pediatric dental hygiene education [9,10,11,12,13]. Despite these advancements, healthcare providers face challenges related to data management and potential biases when using AI-based solutions. However, recent studies show promising results in reducing both diagnostic time and associated costs [14].

Machine learning and computer vision have steadily gained traction in efforts to detect various oral diseases [15,16,17,18,19,20,21,22,23]. For instance, deep learning models applied to microscopic intraoral images facilitate periodontal disease and bone loss diagnosis [24]. Similarly, certain dental treatments and appliances can lead to cavity formation and plaque buildup, illustrating the need for a proactive approach to detection [25]. These diagnostic and treatment hurdles are often pronounced in emerging economies such as Mexico, where resource constraints underscore the importance of cost-effective, widely accessible methods. In our study, we focus on using diverse mobile devices (e.g., smartphones from Apple Inc., Cupertino, CA, USA or Samsung Electronics Co., Suwon, Republic of Korea) to acquire data for detecting and preventing oral diseases.

Although AI has ushered in transformative possibilities in dentistry, challenges persist due to limited data availability, interpretability issues, lack of transparency, and class imbalance [26]. Indeed, dentists have used infrared and X-ray technologies since the 1950s to diagnose dental conditions [22], but the need for more user-friendly and efficient diagnostic technologies remains pressing. Many existing studies make use of datasets spanning between 88 and 12,600 clinical images.

Deep learning models are now central to accelerating and enhancing dental procedures. Consequently, it is crucial to assess the accuracy and efficacy of such models in dental plaque detection. Differentiating healthy dental plaque from plaque affected by periodontitis using neural networks has proven useful for clinical diagnosis, particularly when working with microscopic plaque images [27].

Several contemporary approaches adopt transformer-based AI, rely on local and global feature segmentation, or incorporate self-attention modules to achieve intelligent segmentation of the dental plaque, and even facilitate tooth identification and numbering [28,29,30,31,32]. In many of these works, researchers employ publicly available deep learning frameworks such as PyTorch (version 1.13.1, Meta AI, Menlo Park, CA, USA) or TensorFlow (version 2.12, Google LLC, Mountain View, CA, USA), as well as YOLO-based object detection systems (e.g., YOLOv3–YOLOv11 [2,3,4,5]), which are accessible online (see https://pjreddie.com/darknet/yolo, accessed on 28 December 2024). Where exact version numbers are not provided in the original references, researchers often specify the repository URL and the date on which they accessed the code.

Because plaque control is complicated without consistent professional monitoring, systematic data collection and labeling are essential for classifying dental surfaces as diseased, healthy, or uncertain [33]. This enables early detection of gingivitis triggered by plaque accumulation.

The application of image processing algorithms for plaque detection and quantification shows a high correlation with manual evaluation, indicating that such automation substantially enhances dental workflows [34]. Both 2D and 3D data offer benefits; while 2D imaging clearly showcases plaque presence on flat surfaces, 3D data from intraoral scanners (e.g., TRIOS^®^ by 3Shape, Copenhagen, Denmark) provide access to difficult-to-reach areas [35].

Deep learning models trained on primary teeth images stained with plaque-disclosing agents (such as Mira-2-Ton^®^, Hager & Werken GmbH & Co. KG, Duisburg, Germany) have exhibited high accuracy in identifying plaque areas [36]. Likewise, when evaluating deep learning-based plaque identification on permanent teeth, results have been clinically acceptable and broadly consistent with dentists’ assessments [37]. Certain studies compare different computer vision algorithms, revealing that using neural networks and leveraging the three RGB channels yields superior performance, especially when utilizing red fluorescent plates as labels [38].

Finally, smartphones are now ubiquitous and are increasingly used in healthcare for data acquisition, screening, and preliminary diagnosis [39]. Such devices enable point-of-care imaging, facilitating the remote assessment of various oral conditions. As the subsequent sections will detail, our proposed approach leverages these widely available mobile devices to detect and monitor plaque in a cost-effective manner, aiming to improve oral healthcare outcomes in diverse settings.

## 3. Methods

The proposed method allows for the detection of new, mature, and over-mature dental plaques to prevent potential oral diseases, automating detection and expediting the identification of the most dangerous plaque (over-mature), often associated with a spectrum of dental pathologies. These types of plaque occur because the mouth is the main entry point for bacteria into the body. Oral hygiene and related habits contribute to the development of oral diseases, which could potentially be life-threatening.

**Figure 4:**
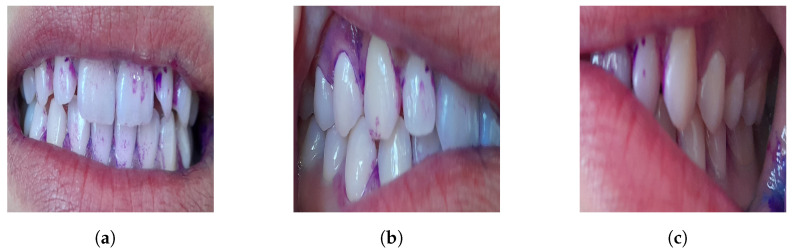
Occlusion in data acquisition: (**a**) frontal occlusion, (**b**) left occlusion, and (**c**) right occlusion.

Plaque, which is a colorless substance formed by proteins in saliva that adhere to teeth and gums, causes diseases that can be prevented through timely diagnosis and intervention. Mobile devices are used to detect dental plaque. Three different images are captured for each sampled individual. Figure 4 illustrates the three views in which the data were collected: frontal occlusion (a), left occlusion (b), and right occlusion (c).

We propose a dataset of RGB images captured via various mobile devices, which we segment, normalize, and then classify into three distinct plaque categories. Our approach significantly improves plaque detection accuracy. Through these methods, our goal is to raise public awareness and propose a methodology that generalizes well across data with wide variability. We assembled a dataset of 531 oral images of people aged five and older in the central zone of Mexico, predominantly from suburban areas. We employ MixUp and CutMix techniques to strengthen data robustness and ensure better generalization during model training.

**Figure 5:**
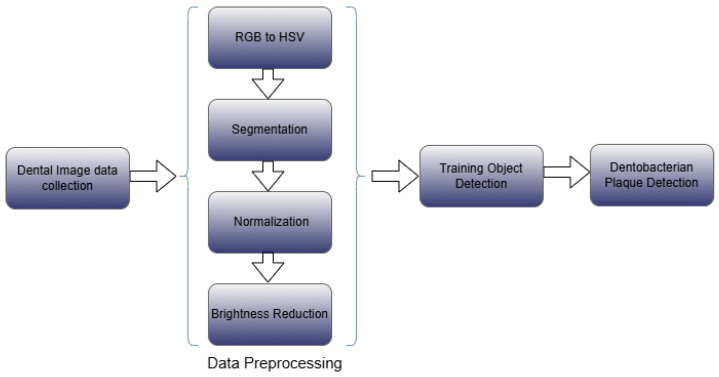
The methodology consists of four stages: (1) data acquisition and labeling, (2) preprocessing, (3) training and evaluation, (4) plaque accumulation assessment.

Figure 5 illustrates the proposed methodology, which consists of four stages, described as follows:

1. Data acquisition: In this stage, we created a dataset of dental images and labeled them with three types of dental plaque—new, mature, and over-mature.
2. Preprocessing: To accurately detect dental plaque, we labeled the images and implemented four preprocessing steps:
  (a) Conversion from RGB to HSV: We transformed the original images, captured in RGB format, into the HSV color space to facilitate segmentation based on specific hues, saturation, and values.
  (b) Segmentation: We identified and removed lip regions, eliminating irrelevant areas for plaque analysis.
  (c) Brightness reduction: We suppressed excessive brightness in the images to minimize the effects of lighting variations, improving data quality for subsequent stages.
  (d) Color normalization: We adjusted the colors in the images to maintain chromatic consistency and enhance comparability between samples.
3. Training and evaluation: We trained and evaluated the three most recent YOLO architectures—YOLOv9 [3], YOLOv10 [4], and YOLOv11 [5]—to detect the three types of plaque, focusing on dental and gingival textures.
4. Results comparison: In this final stage, we compared the results obtained from the three models and used the O’Leary index to evaluate plaque accumulation.

### 3.1. Data Acquisition

To collect the RGB data, we used ten different mid- and low-range mobile devices. We sampled the data in the central area of Mexico, mainly in the suburban and urban areas of León, Silao, Celaya, Comonfort, Cortazar, Juventino Rosas, and Apaseo el Grande in the state of Guanajuato. We applied a revealing gel to each sample to identify the type of plaque: pink color indicates new plaque, purple indicates mature plaque, and blue indicates over-mature plaque. In total, we obtained RGB images from 177 individuals, which allowed the collection of oral data, totaling 531 RGB images of dental plaque. The obtained images were scaled to the lowest resolution presented by the mobile devices of 1280 × 1280 pixels. Then, we manually annotated dental plaque into three categories: new, mature, and over-mature.

### 3.2. Data Preprocessing

The detection of dental plaque by color aims to identify specific regions in an image that correspond to dental plaque within defined color ranges. It utilizes the HSV (Hue, Saturation, Value) color model for segmentation based on chromatic features.

#### 3.2.1. Segmentation

The segmentation process aims to extract only areas containing dental plaque, suppressing irrelevant information that could interfere with analysis. A significant challenge is the unavoidable presence of lips in the images captured during data collection.

**Figure 6:**
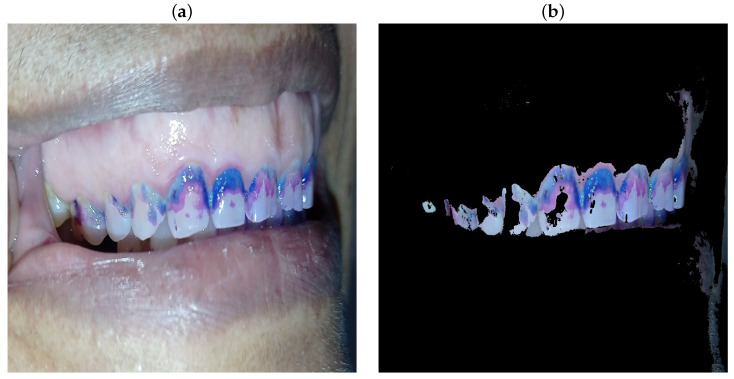
Segmentation: Comparison between the original image and the segmented image for dental plaque detection. (**a**) shows the original image of an oral region showing teeth with dental plaque revealed using an indicator gel. The plaque appears in blue and purple tones adhered to the tooth surfaces, particularly in interdental contact areas and along the gingival margin. (**b**) shows the segmented image processed in the HSV color space. Segmentation removes irrelevant information, such as lips and background, and highlights only areas with dental plaque. The segmented regions display the same blue and purple tones, enabling clear identification and precise quantification of the plaque.

In many cases, the lip area may also contain residues of the disclosing gel, introducing false negatives into the detection process. This interference reduces accuracy, as regions that are not related to dental plaque can be mistakenly considered relevant or incorrectly discarded. Figure 6 shows an image captured with a revealing gel for the detection of dental plaque. The image on the left highlights three distinct shades that indicate different stages of plaque development: pink for new plaque, purple for mature plaque, and blue for over-mature plaque. The image on the right shows the segmentation applied to suppress the lip region, highlighting only the areas corresponding to dental plaque.

#### 3.2.2. Brightness Reduction and Normalization

Once the areas of interest are segmented and the average color ranges in the HSV color space are obtained from the dataset, the process involves calculating the global averages of white color saturation and normalizing the three types of dental plaque. This procedure improves efficiency in plaque detection, considering that the images were captured using different mobile devices and under various lighting conditions.

First, we reduce the brightness of the image $I_{\mathrm{HSV}}$ when the brightness is greater than a pre-computed threshold T=230. Then, we calculate the global average values of saturation and the white color value using Equation (1).

(1) $$s_{\mathrm{avg}}=\frac{1}{N}\sum_{i=1}^{N}S_{i},\;v_{\mathrm{avg}}=\frac{1}{N}\sum_{i=1}^{N}V_{i},$$

where *S* is the saturation channel, *V* is the value channel, and *N* is the total number of pixels. For the colors of the dental plaque (pink, purple, blue), calculate the global averages of the lower and upper limits using Equation (2).

(2) $$L_{\mathrm{avg}}=\frac{1}{N}\sum_{i=1}^{N}L_{i},\;U_{\mathrm{avg}}=\frac{1}{N}\sum_{i=1}^{N}U_{i},$$

where $L_{\mathrm{avg}}$ is the global average of the lower bounds for the specified color ranges (e.g., pink, purple, blue). $U_{\mathrm{avg}}$ is the global average of the upper bounds for the specified color ranges. *N* the total number of pixels or samples considered for calculating the averages. $L_{i}$ is the lower bound value of the *i*-th pixel or sample in the specific color range. $U_{i}$ is the upper bound value of the *i*-th pixel or sample in the specific color range. $\sum_{i=1}^{N}$ aggregates the values across all *N* pixels or samples.

Finally, the global averages and the threshold T are applied to the image $I_{\mathrm{HSV}}$, obtaining the normalization of each image.

The goal of normalizing white color is to ensure consistency and uniformity in image lighting and color, suppressing white areas corresponding to teeth. This step is crucial because of the variations that can arise during data acquisition, such as lighting, brightness correction, chromatic reference, and the reduction in false positives and negatives.

The average saturation and value specifically for the white color are calculated because white has unique characteristics in the HSV space. In the Hue (H) channel, white is not well defined since it lacks a dominant tone. In the saturation (S) channel, white has values near zero, indicating that the color is “desaturated.” Finally, the value (V) channel measures the brightness intensity, which tends to be high for white.

The average values are obtained to achieve consistency in the S and V channels, providing a global representation of variations in brightness and intensity for the white color. Normalizing white allows adjustment of the V intensity, reducing the effects of uneven lighting conditions and ensuring consistency in white regions across all processed images.

Normalization of the lower and upper limits across different images unifies variability in tone and saturation caused by changes in capture conditions (e.g., lighting and camera settings). Averaging these limits smooths the variations and produces representative ranges. During color normalization, these global ranges are used as a reference to adjust and maintain tonal consistency in the images.

$$\mathrm{Normalized}=\left[\begin{array}{c} Pink\\ 120\\ 200\\ 200 \end{array}\right],\;\left[\begin{array}{c} Purple\\ 125\\ 200\\ 200 \end{array}\right],\;\left[\begin{array}{c} Blue\\ 100\\ 200\\ 200 \end{array}\right],\;\left[\begin{array}{c} White\\ 0\\ 0\\ 255 \end{array}\right]\;$$

**Figure 7:**
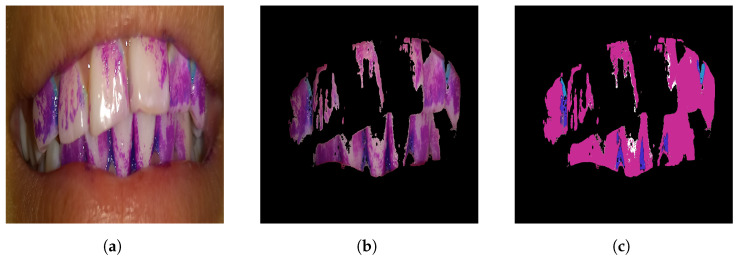
Brightness removal and normalization. (**a**) shows the original image in RGB format with a disclosing gel applied to highlight the presence of plaque. (**b**) displays the image transformed into the HSV color space, where the brightness component is removed, and specific saturation and value ranges are suppressed. (**c**) shows the resulting image showing the three types of dental plaque normalized and free from brightness interference, improving the precision of plaque detection.

The results are expressed in the normalized vector for the three types of plaque—new, mature, and over-mature—as well as for white. Figure 7 illustrates this process. In (a), the original image in RGB format shows plaque highlighted by a disclosing gel; (b) shows the image transformed into the HSV color space, where the brightness component is removed, and the average saturation and value for the white color are calculated, suppressing values corresponding to dental surfaces; finally, (c) shows the resulting image with the three types of dental plaque normalized and free from brightness and tooth interferences, which significantly improves the precision of plaque detection.

### 3.3. Detection of Dentobacterial Plaque

Dental plaque is an accumulation of microorganisms that can cause visible discoloration on teeth and gums and is associated with the development of oral diseases. While dentists can visually identify its presence, the use of disclosing gels is essential for classifying specific types of plaque. These gels highlight the characteristic color changes of each type of plaque, facilitating both its analysis and the design of more precise treatments.

In this study, the three most recent versions of YOLO were implemented to detect and classify the color changes associated with dental plaque. YOLO is an advanced object detection model characterized by its high efficiency and accuracy, making it an ideal tool for automated diagnostic applications in dentistry.

In [40], YOLO’s operation is described as being based on three main stages: single-step grid predictions, bounding box regression, and non-maximum suppression (NMS). The model’s main advantages include its speed, enabling real-time detection using standard hardware, and its ability to utilize global image features. However, disadvantages, such as a lower likelihood of predicting false positives and difficulty detecting small objects, are also noted.

This work focuses on comparing the three most recent versions of YOLO: YOLOv9 [3], YOLOv10 [4], and YOLOv11 [5]. These versions propose significant architectural improvements aimed at overcoming previous limitations, optimizing both accuracy and efficiency. The features of each version are briefly described in the following sections.

### 3.4. Evaluation Metrics

In order to evaluate the performance of the dental plaque detection task, we compared the predicted bounding boxes (i.e., detections) against the ground truth bounding boxes for each image. Specifically, we counted all bounding boxes in both the ground truth and detection results, assigning each predicted box to its best-matching ground truth box based on the highest Intersection over Union (IoU) score. A predicted box was considered a true positive (TP) if its IoU with a particular ground truth box exceeded a predefined threshold (e.g., 0.5). Any predicted box not matching a ground truth box above this threshold was regarded as a false positive (FP). Conversely, any ground truth box not matched by a predicted box above the threshold was deemed a false negative (FN). The true negatives (TNs) are implied but typically not used in the context of object detection metrics.

After aggregating the TPs, FPs, and FNs across all images, we calculated the following metrics:

#### 3.4.1. Precision

Precision measures the ratio of correctly predicted positive detections to the total predicted positives, as defined in Equation (3):

(3) $$\mathrm{Precision}=\frac{TP}{TP+FP}.$$

#### 3.4.2. Recall

Recall evaluates the ratio of correctly predicted positive detections to the total number of ground truth objects, as given in Equation (4):

(4) $$\mathrm{Recall}=\frac{TP}{TP+FN}.$$

#### 3.4.3. Mean Average Precision (mAP)

The mean average precision (mAP) combines precision and recall into a single metric to comprehensively evaluate detection performance. First, the average precision (AP) is computed for each class by measuring the area under the precision–recall curve. The mAP score is then obtained by averaging the AP across all object classes and, if applicable, multiple IoU thresholds. A higher mAP score indicates that the model achieves both high precision (i.e., accurate detections) and high recall (i.e., comprehensive coverage of all objects), which is crucial for reliable and robust performance in real-world applications.

#### 3.4.4. O’Leary Index

To evaluate the accumulation of dental plaque on tooth surfaces in the analyzed images, we use the O’Leary index [41]. Each tooth was divided into four areas (cervical, middle, incisal or occlusal, and lingual or vestibular), and the presence of plaque in each region was assessed. The O’Leary index is computed according to Equation (5):

(5) $$IL=\frac{N_{APC}}{N_{TAE}}\times 100,$$

where $N_{APC}$ represents the number of dental areas affected by plaque and $N_{TAE}$ is the total number of areas examined on the teeth. A higher O’Leary index value indicates a greater presence of plaque on the tooth surfaces under analysis.

## 4. Results

The proposed approach aims to detect three categories of dental plaque: new, mature, and over-mature. Since older plaque poses higher health risks, achieving accurate detection for mature and over-mature plaque is a primary clinical concern. We evaluated three state-of-the-art YOLO variants (YOLOv9, YOLOv10, and YOLOv11) in detecting these plaque categories. All models were trained on the same annotated dataset, which includes 431 images for training and 100 images for validation as well as bounding box labels for the three distinct plaque classes.

### 4.1. Performance Overview Across YOLO Versions

We tested multiple scaled variants within each YOLO version to identify the one offering the best trade-off between accuracy and efficiency. Specifically, we explored t, s, m, c, e in YOLOv9; n, s, m, b, l, x in YOLOv10; and n, s, m, l, x in YOLOv11. Table 1 summarizes the results in terms of Precision, Recall, and mAP@50 (mean Average Precision at a 50% IoU threshold). For each YOLO variant, we highlight the best-performing scale in bold.

Among all YOLOv9 scales, YOLOv9s attained the highest mAP@50 (0.680). In the YOLOv10 family, YOLOv10m emerged as the best performer with a 0.686 mAP@50. Meanwhile, YOLOv11m exhibited the top overall performance, recording a 0.713 mAP@50. These results indicate that, although all YOLO versions can detect plaque, more recent versions (YOLOv10 and YOLOv11) exhibit higher overall accuracy and recall.

### 4.2. Class-Wise Analysis (New, Mature, Over-Mature)

**Figure 8:**
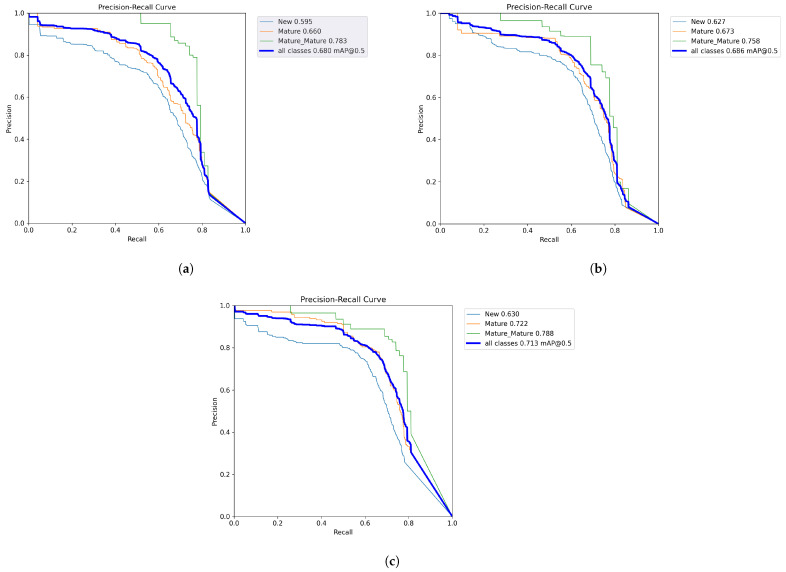
Precision–recall curves for the three best-scoring YOLO models on the validation set: (**a**) YOLOv9s, (**b**) YOLOv10m, and (**c**) YOLOv11m. Each color-coded line depicts a plaque class (blue for new, orange for mature, green for over-mature), and the mean AP at IoU = 0.5 is shown for each. YOLOv11m achieves superior performance in detecting both mature and over-mature plaque.

A deeper understanding of each model’s behavior is critical for evaluating the capacity to detect plaque “age.” Hence, we tracked each class’s Average Precision (AP) within YOLOv9s, YOLOv10m, and YOLOv11m, the respective top performers of each YOLO version. Figure 8 illustrates the precision–recall curves for these three best-performing models:

- YOLOv9s (Figure 8a):
  – New Plaque (pink): AP ≈59.5%.
  – Mature Plaque (purple): AP ≈66.0%.
  – Over-Mature (blue): AP ≈78.3%.
  While YOLOv9s generally performed well across classes, its detection of new plaque was more susceptible to lighting and color variations, occasionally confusing faint pink areas with the background.
- YOLOv10m (Figure 8b):
  – New Plaque (pink): AP ≈62.7%.
  – Mature Plaque (purple): AP ≈67.3%.
  – Over-Mature (blue): AP ≈75.8%.
  By introducing dual label assignments and a more efficient path aggregation network, YOLOv10m boosted consistency in plaque localization. The model showed modest improvement for new plaque compared to YOLOv9s and comparable results for mature and over-mature plaque.
- YOLOv11m (Figure 8c):
  – New Plaque (pink): AP ≈63.0%.
  – Mature Plaque (purple): AP ≈72.2%.
  – Over-Mature (blue): AP ≈78.8%.
  YOLOv11m showed the best precision–recall balance, particularly in identifying mature plaque (purple). Enhanced feature extraction modules and attention mechanisms reduced confusion between mature and over-mature classes, leading to a higher overall mAP@50.

### 4.3. Qualitative Examples of Plaque Detection

**Figure 9:**
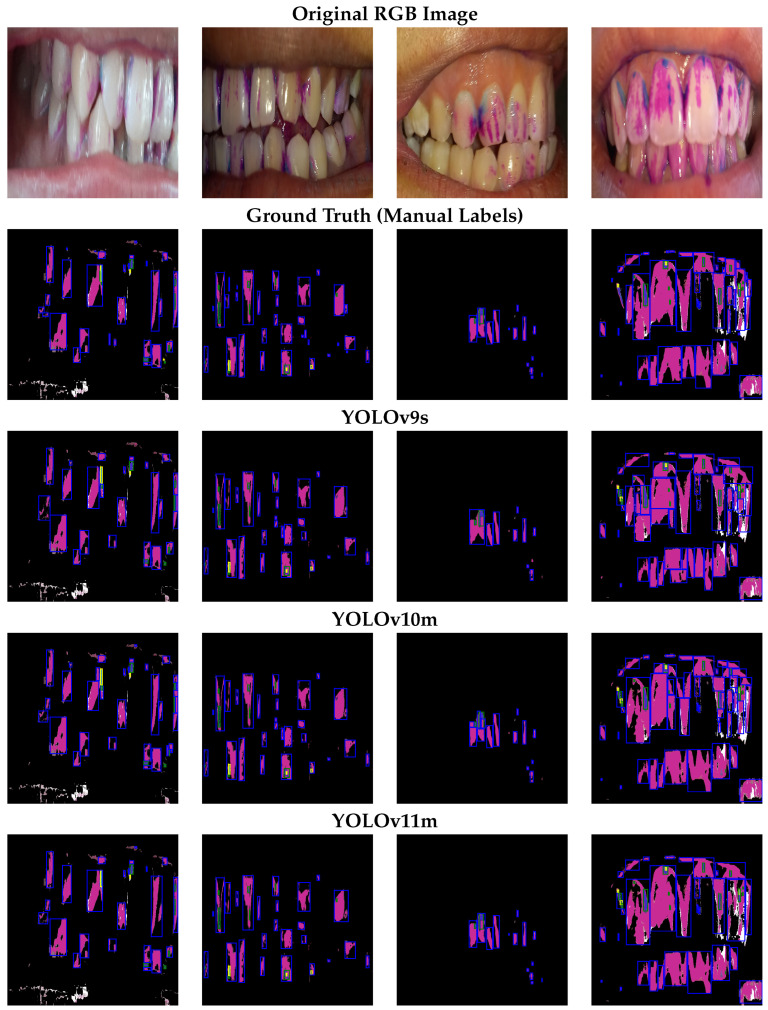
Qualitative examples of dental plaque detection. Each column shows one patient sample across different models. The ground truth row (manual annotation) highlights new plaque in blue, mature plaque in green, and over-mature plaque in yellow.

Figure 9 shows how YOLOv9s, YOLOv10m, and YOLOv11m detect plaques in several sample images. The first row presents the original RGB images, and the second row illustrates the ground truth bounding boxes. The subsequent three rows correspond to predictions by YOLOv9s, YOLOv10m, and YOLOv11m.

All three detectors consistently identify the most prominent areas of plaque. YOLOv9s occasionally underestimates small patches of new plaque near the gingival margin, whereas YOLOv10m and YOLOv11m tend to better detect subtle color differences. YOLOv11m often offers bounding boxes more closely aligned with ground truth annotations, reflecting its stronger learned representations.

### 4.4. Statistical Distribution of Plaque Size and O’Leary Index Analysis

**Figure 10:**
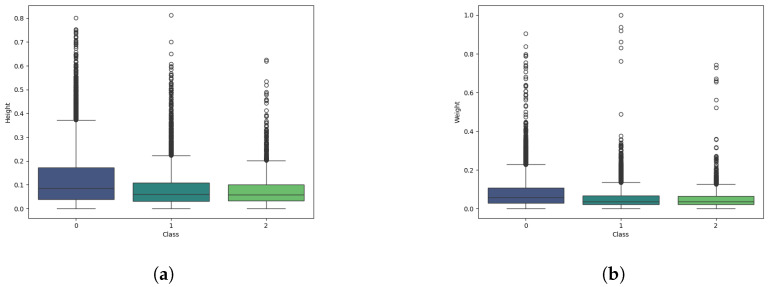
Distribution of dental plaque bounding boxes by (**a**) height and (**b**) width, normalized to the [0, 1] interval. Classes: 0 = new plaque, 1 = mature plaque, 2 = over-mature plaque. Outliers show that some images contain substantial plaque regions.

To further assess detection robustness, we examined the distribution of plaque bounding box sizes. Figure 10 shows box plots for the normalized width and height of ground truth boxes across the three classes. Most plaques are relatively small (reflecting early or mild accumulations), but outliers representing larger contiguous plaque areas are also observed.

**Figure 11:**
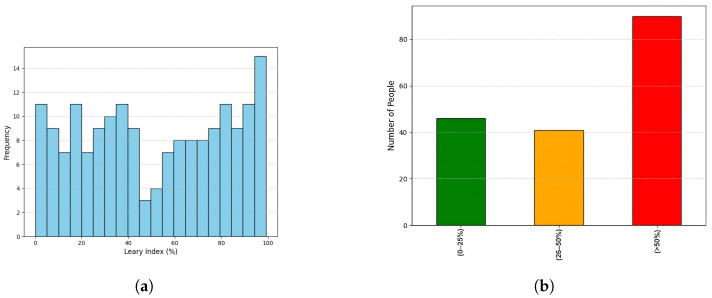
(**a**) Histogram of O’Leary index across 177 samples, showing a broad distribution from minimal to severe plaque accumulation. (**b**) Breakdown of O’Leary index into Mild, Moderate, and Severe categories, highlighting that a significant portion of the study population suffers from extensive plaque accumulation (>50%).

Beyond detection metrics, we also analyzed plaque severity using the O’Leary index. Figure 11a displays a histogram of O’Leary index values for 177 individuals, while Figure 11b categorizes the results as:

- Mild (0–25%);
- Moderate (26–50%);
- Severe (>50%).

Over 80 individuals fell into the severe category, indicating a high plaque burden that poses a significant health risk. Although the O’Leary index does not directly affect the precision or recall of models, it complements our pipeline by summarizing the prevalence of plaque and contextualizing it clinically.

### 4.5. Discussion of Plaque Age Detection

Our comparative study demonstrates that YOLOv11m offers the most robust detection overall, especially for mature (purple) and over-mature (blue) plaque. This aligns with architectural improvements introduced in YOLOv11, including refined backbone/neck modules and improved attention mechanisms. It is especially advantageous in differentiating shades of purple and blue, which can be visually similar under varied lighting.

All YOLO versions generally detect over-mature plaque more accurately than new plaque. This is likely because:

- Over-mature plaque (blue) often forms larger, denser clusters, presenting more visually prominent regions.
- New plaque (pink) can appear faint and at times blend with gum tissue, increasing the risk of false negatives.

In summary, YOLOv9s and YOLOv10m already provide a solid performance baseline; however, YOLOv11m consistently registers higher AP values for all three classes, reflecting its adaptability to the diverse color intensities and lighting variations present in our dataset. This capacity to discriminate small color gradations is crucial for correct “plaque age” identification and could significantly aid dentists in diagnosing patients at early or high-risk stages.

## 5. Discussion and Conclusions

This study aimed to automate the detection of three dental plaque types—new, mature, and over-mature—using modern YOLO architectures applied to RGB images captured with mobile devices under varying lighting conditions. The key finding is that the YOLOv11m model demonstrated the highest detection performance and best class-wise discrimination among the tested architectures, confirming the feasibility of deep learning-based plaque detection for real-world clinical or public health scenarios. Compared to conventional methods reliant on manual visual assessments or indicator dyes [33,34], our work adds a time-dependent dimension by discriminating among plaque ages, which is clinically relevant because older plaque correlates with progressive diseases such as gingivitis and periodontitis [8].

A notable advantage of our approach is the flexible data acquisition process, in which standard mid-range and low-range mobile devices were used to collect images, reflecting real-world constraints faced by lower-resource settings. The World Health Organization (WHO) has highlighted the importance of making oral health screenings widely accessible [1], and this method meets that need by reducing reliance on specialized hardware. Furthermore, our robust preprocessing strategy (involving segmentation, brightness normalization, and color consistency) substantially boosted the performance of YOLO models, mitigating the impact of imaging artifacts and environmental fluctuations that are common when using smartphones.

An important clinical benefit is the heightened sensitivity for detecting mature and over-mature plaque, which is especially significant given that older plaque often leads to more severe oral pathologies requiring immediate attention. While all YOLO variants generally excelled at identifying these stages, they showed more variability in detecting new plaque, likely because faint pink tones can merge with the gum tissue or be overshadowed by lighting conditions. By incorporating the O’Leary index into our pipeline, we also provided a clinically interpretable measure of plaque severity. This index aggregated the detected plaque into mild, moderate, or severe categories, aligning our automated analysis with a standard dental plaque scoring system. Integrating an objective index such as the O’Leary index helps clinicians quantify disease burden more consistently than subjective visual examinations alone.

Despite these promising findings, there are a few limitations. First, although our dataset comprises over 500 images from multiple mobile devices, it remains smaller than large-scale medical image repositories, which limits statistical power and model generalizability. Second, data collection took place in the central region of Mexico, so socio-environmental factors (diet, oral hygiene habits, and healthcare infrastructure) may differ from other settings, potentially affecting plaque characteristics. Third, while YOLO automates plaque detection, image acquisition still relies on a cooperative patient or a trained practitioner for proper device positioning, which can introduce variability. Finally, staining with a disclosing gel, although standard in many dental offices, may not always be practical in community screenings or self-assessment scenarios without professional help.

Future work should address these points by expanding the dataset to include broader demographic and geographical diversity, thereby enhancing the robustness of the model to varied oral health conditions. An attractive next step is developing a real-time mobile application that can provide on-the-spot feedback on plaque accumulation, potentially guiding users in their daily oral hygiene routines and supporting teledentistry in underserved regions. In addition, future research might incorporate 3D imaging or advanced modalities to capture plaque in regions that are more difficult to reach with conventional 2D photography, such as posterior teeth or interproximal areas. Another direction could involve multi-task learning for detecting additional dental pathologies, like caries or gum recession, in tandem with plaque detection.

Overall, our findings confirm that advanced YOLO architectures can accurately detect and classify plaque ages, and that preprocessing steps tailored to smartphone images significantly enhance the robustness and precision of detection. This framework suggests an avenue for practical, cost-effective oral healthcare tools that can adapt to variable imaging conditions and socio-economic contexts. By reducing the reliance on subjective visual examination, our automated approach holds promise for earlier intervention, more standardized tracking of dental health, and potential cost savings for both patients and health systems. We anticipate that continued research and widespread validation of this method, particularly in multi-center or international collaborations, will further advance the role of artificial intelligence in improving global oral health outcomes.

## Figures and Tables

**Table 1:** Comparison of model accuracy across YOLO versions. Precision, recall, and mAP@50 are reported for models trained to detect new, mature, and over-mature plaque. Each row corresponds to a YOLO variant at a particular scale (t, s, m, c, e, n, b, l, x, etc.). The best-performing scales per YOLO version are shown in bold.

Detector	Model	Precision	Recall	mAP50
YOLOv9	t	0.725	0.625	0.649
	**s**	**0.761**	**0.616**	**0.680**
	m	0.704	0.627	0.653
	c	0.697	0.644	0.657
	e	0.675	0.616	0.647
YOLOv10	n	0.686	0.604	0.612
	s	0.733	0.608	0.661
	**m**	**0.758**	**0.650**	**0.686**
	b	0.756	0.645	0.662
	l	0.715	0.688	0.667
	x	0.761	0.640	0.684
YOLOv11	n	0.702	0.578	0.642
	s	0.723	0.618	0.650
	**m**	**0.794**	**0.654**	**0.713**
	l	0.768	0.636	0.693
	x	0.700	0.642	0.660

## Data Availability

Dataset available on request from the authors.
